# Polycythemia vera: historical oversights, diagnostic details, and therapeutic views

**DOI:** 10.1038/s41375-021-01401-3

**Published:** 2021-09-03

**Authors:** Ayalew Tefferi, Alessandro M. Vannucchi, Tiziano Barbui

**Affiliations:** 1 Division of Hematology, Department of Medicine, Mayo Clinic, Rochester, MN USA; 2 Department of Experimental and Clinical Medicine, CRIMM, Center Research and Innovation of Myeloproliferative Neoplasms, Azienda Ospedaliera Universitaria Careggi, University of Florence, Florence, Italy; 3 Research Foundation, Papa Giovanni XXIII Hospital, Bergamo, Italy

**Keywords:** Myeloproliferative disease, Myeloproliferative disease

## Abstract

Polycythemia vera (PV) is a relatively indolent myeloid neoplasm with median survival that exceeds 35 years in young patients, but its natural history might be interrupted by thrombotic, fibrotic, or leukemic events, with respective 20-year rates of 26%, 16%, and 4%. Current treatment strategies in PV have not been shown to prolong survival or lessen the risk of leukemic or fibrotic progression and instead are directed at preventing thrombotic complications. In the latter regard, two risk categories are considered: high (age >60 years or thrombosis history) and low (absence of both risk factors). All patients require phlebotomy to keep hematocrit below 45% and once-daily low-dose aspirin, in the absence of contraindications. Cytoreductive therapy is recommended for high-risk or symptomatic low-risk disease; our first-line drug of choice in this regard is hydroxyurea but we consider pegylated interferon as an alternative in certain situations, including in young women of reproductive age, in patients manifesting intolerance or resistance to hydroxyurea therapy, and in situations where treatment is indicated for curbing phlebotomy requirement rather than preventing thrombosis. Additional treatment options include busulfan and ruxolitinib; the former is preferred in older patients and the latter in the presence of symptoms reminiscent of post-PV myelofibrosis or protracted pruritus. Our drug choices reflect our appreciation for long-term track record of safety, evidence for reduction of thrombosis risk, and broader suppression of myeloproliferation. Controlled studies are needed to clarify the added value of twice- vs once-daily aspirin dosing and direct oral anticoagulants. In this invited review, we discuss our current approach to diagnosis, prognostication, and treatment of PV in general, as well as during specific situations, including pregnancy and splanchnic vein thrombosis.

## Historical prelude

Polycythemia vera (PV), “maladie de Vaquez,” was first described by Louis Henri Vaquez (1860–1936), a French physician, in 1892 [1]. A few additional cases were later described and systematically reviewed by William Osler (1849–1919) in 1903 [2]. In 1951, William Dameshek (1900–1969) included PV in his conceptual classification of myeloproliferative disorders, now referred to as “myeloproliferative neoplasms (MPN),” along with essential thrombocythemia (ET) and primary myelofibrosis (PMF) [3]. Dameshek’s concept of MPN was genetically ratified in 2005 by the seminal discovery, across these three clincopathologic entities, of a *JAK2* gain of-function mutation (*JAK2*V617F; a G to T somatic mutation at nucleotide 1849, in exon 14, resulting in the substitution of valine to phenylalanine at codon 617) [4–7]. In 2007, additional *JAK2* mutations in exon 12 were described in *JAK2*V617F-negative patients with PV [8]; *JAK2* mutational frequencies, in PV, are estimated at 97% for *JAK2*V617F and 3% for other *JAK2* mutations, including *JAK2* exon 12. In other words, for all practical purposes, the presence of a *JAK2* mutation is now expected in virtually all patients with PV, a fact that has greatly complemented our morphologic-based diagnostic approach; current literature suggests similar outcome in patients with *JAK2* exon 14 vs exon 12 mutations [9–11]. Laboratory studies examining the pathogenetic role of *JAK2* mutations are highlighted by its origin at the stem cell level and the demonstration of heightened JAK-STAT activation and induction of mutant *JAK2*-driven PV phenotype in mice [5, 12, 13]. *JAK2*V617F is one of three MPN-specific driver mutations that include *CALR* and *MPL* mutations; the latter are usually not found in patients with PV but are prevalent in *JAK2*V617F-negative ET and PMF. It is currently assumed that the phenotypic differences between PV and the other two MPN variants are in part contributed by differences in the specific cytokine receptors that are activated by the corresponding driver mutation and interactions with other co-occurring mutations and their order of acquisition [12].

The historical account of PV therapeutics spans over a century, annotated by key contributions from the Polycythemia Vera Study Group (PVSG), founded in 1967 [14]. The pre-PVSG era included mostly ineffective and potentially detrimental treatment modalities, save for therapeutic phlebotomy [15, 16], including skeletal radiation therapy (1917) [17], acetylphenylhydrazine (1918) [18], potassium arsenite (1933) [19], radiophosphorus (P32) (1940) [20], lead acetate (1942) [21], nitrogen mustard (1950) [22], triethylene melamine (1952) [23], pyrimethamine (1954) [24], busulfan (1958) [25], 6-mercaptopurine (1962) [26], pipobroman (1962) [27], uracil mustard (1964) [28], chlorambucil (1965) [29], and dapsone (1966) [30]. Hydroxyurea (HU) and melphalan were added to the list in 1970 [31, 32]. Early retrospective studies in PV had suggested a superior median survival with myelosuppressive therapy as opposed to either no treatment (median survival ∼18 months) or treatment with phlebotomy alone (median survival close to 4 years) [33], while at the same time raised concerns regarding myelosuppressive drug leukemogenecity [34, 35]. The PVSG clinical trials, shepherded by Louis Wasserman (1912–1999), were designed to clarify these issues at hand with support from NIH that lasted until 1987 and included 14 separate studies [36]. The PVSG studies implicated both chlorambucil and P32, but not HU, as being leukemogenic and detrimental to survival [37, 38], although the leukemogenic hazards of HU are still being debated. Across the Atlantic, Tiziano Barbui (1938) and fellow investigators from Europe have successfully conducted a series of controlled prospective studies that have confirmed the antithrombotic value of keeping the hematocrit target below 45%, with phlebotomy ± HU/cytoreductive therapy (2013) [39], and low-dose aspirin therapy (2004) [40] in PV, and that of HU in high-risk ET (1995) [41]. The expanding therapeutic armamentarium for PV now includes pegylated interferon (peg-INF) [42] and ruxolitinib [43]. Over the last several years, we have been involved in the development of both the 2008 [44] and 2016 [45] World Health Organization (WHO) classification system for MPNs and have in addition fostered contemporary diagnostic and treatment algorithms [46–49]. In the current review, we considered new developments and also revisited with ongoing controversies in order to outline our current approach in the diagnosis, prognostication and treatment of PV.

## Our current diagnostic approach in polycythemia vera

**Fig. 1:**
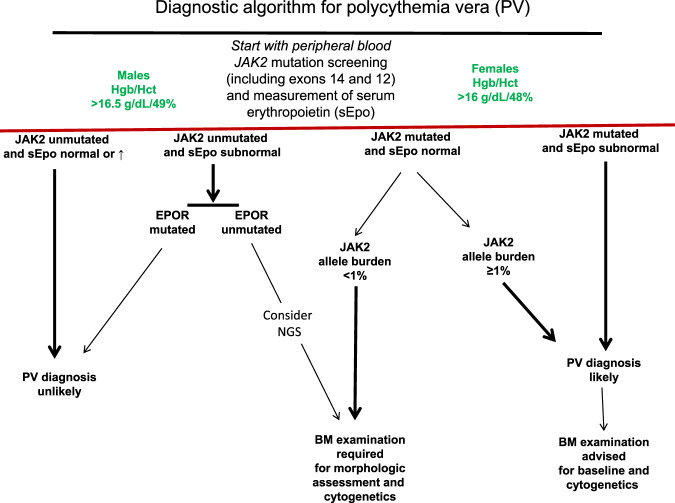
Current diagnostic algorithm for polycythemia vera. Our approach to diagnosis of polycythemia vera (PV).

PV is currently defined by an acquired increase in hemoglobin/hematocrit level above 16.5 gm/dL/49% in men and 16 g/dL/48% in women, in the context of a *JAK2* mutation and characteristic bone marrow morphology. The 2016 WHO classification system for hematopoietic tumors recognizes the almost perfect association between PV and a *JAK2* mutation, as well as the fact that *JAK2*V617F is also detected in 50–70% of patients with either ET or PMF [45]. The formal diagnostic table lists three major (Hb/Hct level above 16.5 g/dL/49% in men and 16 g/dL/48% in women or red cell mass >25% above mean normal predicted value; consistent bone marrow morphology; and presence of a *JAK2*V617F or exon 12 mutation) and one minor (subnormal serum erythropoietin (Epo) level) criteria; WHO-qualified diagnosis requires the presence of either all three major criteria or the first two major criteria plus the minor criterion [50]. Our current approach to the diagnosis of PV is consistent with these fundamentals, with some modifications that accommodate clinical practice scenarios, which are further elaborated below (Fig. 1). In general, screening for other mutations through next-generation sequencing (NGS) or cytogenetic abnormalities is more useful in terms of prognostication (discussed below in the section of prognosis) rather than diagnosis.

### JAK2 mutation screening

Virtually all patients with PV harbor either *JAK2*V617F (exon 14; 97% sensitivity) or *JAK2* exon 12 mutation (majority of *JAK2*V617F-negative cases) [51]. Accordingly, the first step in approaching the diagnosis of PV should include *JAK2* mutation screening, and we favor upfront targeting of both exons 14 and 12, in order to avoid undue delay in the diagnostic process; it should also be noted that peripheral blood and bone marrow samples are equally informative in detecting and quantifying *JAK2*V617F [52]. In order to address issues with inconclusive test results and also provide an additional layer of diagnostic comfort, we recommend concomitant measurement of serum Epo level, which is expected to be subnormal in more than 85% of patients with PV [53]. *JAK2* mutation screening might also be a more sensitive diagnostic tool, compared to bone marrow morphology, in patients presenting with “MPN-unclassifiable (MPN-U)” phenotype or splanchnic vein thrombosis (SVT), as discussed below [54].

### Is bone marrow examination mandatory for the diagnosis of PV

The official WHO diagnostic criteria for PV allow bypassing bone marrow examination, for diagnostic purposes, in *JAK2*-mutated cases with Hb/Hct level above >18.5 g/dL/55.5% in men and 16.5 g/dL/49.5% in women, with subnormal serum Epo. However, we advise the specific procedure in all patients, save for certain clinical scenarios, not only for confirming the diagnosis but also for purposes of establishing a baseline and obtain prognostically relevant cytogenetic information. Exceptional cases include older patients and those with significant comorbidities, where the additional information from bone marrow examination might not affect treatment decisions or long-term prognostication.

### The concept of masked polycythemia vera and MPN-unclassifiable

There is increasing awareness of patients presenting with SVT associated with a *JAK2* mutation, but not meeting the WHO-listed criteria for PV or other MPN. The WHO classification system defines such presentation as MPN-U, which also includes cases with the so-called masked PV [50]. From a biological standpoint, a documented increase in Hb/Hct from an individual’s baseline, associated with a *JAK2* mutation, should be approached as PV, even if Hb/Hct levels do not cross the WHO-defined diagnostic thresholds; such circumstances should also be considered in distinguishing PV from *JAK2*-mutated ET, where *JAK2*V617F allele burden might provide additional clue (i.e., expected to be higher in the former and often <20% in the latter). Similarly, the dilutional effect of marked splenomegaly might underestimate Hb/Hct levels in some patients. In the end, such details regarding diagnostic accuracy might not influence specific treatment strategies, as long as one errs on the side of keeping the Hct level <45%, in equivocal cases [55].

### The relevance of bone marrow fibrosis in the context of PV

A subset of patients with PV displays variable degree of bone marrow fibrosis at time of diagnosis. Such occurrences do not alter the diagnostic label if the other WHO-defined formal criteria are met. We have in the past investigated the prevalence and prognostic relevance of bone marrow fibrosis at time of initial diagnosis of PV [56, 57]; in one study [57], approximately 14% of 526 patients displayed mostly grade 1 reticulin fibrosis that was associated with increased incidence of palpable splenomegaly and post-PV MF but lower incidence of thrombosis. Another study of 262 patients reported an even higher (48%) incidence of grade 1 reticulin fibrosis and confirmed the association with an increased risk of fibrotic transformation [56]. These observations underline the importance of bone marrow examination at time of diagnosis, which also facilitates detection of abnormal karyotype that has previously been associated with inferior survival in PV [58–60]. On the other hand, diagnosis of post-PV MF requires the presence of ≥grade 2 fibrosis, accompanied by development of progressive splenomegaly, anemia, leukoerythroblastosis, or constitutional symptoms [61].

## Prognostication

### Predicting overall, leukemia-free, and myelofibrosis-free survival

Overall survival in PV and other MPNs is inferior to that of age- and sex-matched general population [60, 62]. Age remains the most important predictor of survival in PV; among 665 Mayo Clinic patients with PV seen between 1967 and 2017, 79 (12%) were ages ≤40 years, 226 (34%) ages 41–60, and 360 (54%) ages >60, with corresponding median survivals of 37, 22, and 10 years [63]. In an international study of 1545 patients with PV, age-independent risk factors for overall survival included leukocytosis, venous thrombosis, and abnormal karyotype [60]. The adverse effect of persistent leukocytosis on disease progression was also underlined in a recent study [64]. In the aforementioned international PV study [60], cumulative risk for leukemic transformation was 2.3% at 10 years and 5.5% at 15 years; risk factors for leukemic transformation included older age, abnormal karyotype, leukocytes ≥15 × 10^9^/L and treatment exposure to pipobroman or P32/chlorambucil, but not to HU or busulfan [60]. Other studies have found *JAK2*V617F allele burden of >50% [65], presence of bone marrow fibrosis at time of diagnosis [56, 57], and persistent leukocytosis [64] to be associated with increased risk of fibrotic transformation. More recent studies in PV have confirmed the adverse and age-independent impact on survival of karyotype, leukocytosis, and certain non-*JAK2* mutations including *SRSF2* and *IDH2* [66, 67]; of note, NGS studies have revealed that over 50% of patients with PV harbor DNA sequence variants/mutations other than *JAK2*, with the most frequent being *TET2* (18%), *ASXL1* (15%), and *LNK* (3%) [67, 68]; combined prevalence of adverse mutations (*SRSF2*, *IDH2*, *RUNX1*, *U2AF1*) for overall, leukemia-free or myelofibrosis-free survival in PV was estimated at 5–10% [67]. These observations have led to the development of an integrated clinical and genetic survival risk model for PV, dubbed as mutation-enhanced international prognostic model-PV (MIPSS-PV) [67]; prognostic variables for overall survival assigned in the MIPSS-PV model were age >67 years (three adverse points), leukocytosis (≥15 × 10^9^/L; two adverse points), abnormal karyotype (one adverse point), and *SRSF2* mutation (two adverse points); accordingly three risk categories were considered: low-risk (0–1 points), intermediate-risk (2–3 points), and high-risk (>3 points) with corresponding median survivals of 24, 13.1, and 3.2 years [67]. We believe that additional prognostic information from NGS and/or karyotype is useful when available but not mandated in routine clinical practice; furthermore, additional studies are needed to validate MIPSS-PV and identify prognostically specific cytogenetic abnormalities [58, 59]. Regarding the latter, in one study of 196 cytogenetically annotated patients with PV, the presence of abnormal karyotype predicted inferior overall, leukemia-free, and myelofibrosis-free survival [59]; however, the number of informative cases with specific cytogenetic abnormalities was too small to further delineate their individual prognostic contribution.

### Thrombosis risk stratification

In general, patients with MPN, across all age groups, are at a higher risk of both arterial and venous thrombosis, compared to matched controls [69]. Treatment-relevant risk stratification in PV is designed to estimate the likelihood of thrombotic complications, which is estimated to occur in approximately 26% of patients followed for a median of 20 years [70]. The original PVSG studies had identified advanced age, history of thrombosis, and treatment with phlebotomy alone as the main risk factors for thrombosis [71]. Accordingly, conventional risk stratification in PV includes two risk categories: high-risk (age >60 years or thrombosis history) and low-risk (absence of both risk factors). Clinical practice in PV has since adopted cytoreductive therapy for the management of high-risk patients and this should be considered when evaluating post-PVSG era studies of risk factor analysis [38]. In that context, a multicenter prospective European Collaborative Low-dose Aspirin Polycythemia Vera (ECLAP) study of 1638 patients with PV confirmed that age >65 years and history of thrombosis remained as the most important risk factors for cardiovascular events in patients receiving contemporary treatment, whereas antiplatelet therapy was more effective than cytoreduction in protection against cardiovascular events [72]. In a more recent rendition of the particular ECLAP study, risk factors for arterial thrombosis included prior arterial event and hypertension, and for venous thrombosis included previous venous event and age ≥65 years [73]. These observations were confirmed by a more recent study, which also identified hyperlipidemia and diabetes as risk factors for arterial events and leukocytosis and major hemorrhage for venous events [74]. The detrimental effect of hypertension to arterial thrombosis was underlined by another study [75]. In patients who have already experienced a first thrombotic event, risk factors for recurrence included age >60 years, and, for arterial thrombosis, leukocytosis at time of first event, in patients younger than 60 years old [76, 77].

Additional pro-thrombotic variables in PV considered over the last three decades include leukocytosis [64, 78–81], *JAK2*V617F allele burden [65, 82] and intensity of phlebotomy [83, 84]. In regard to the latter, one retrospective study suggested an association between intensity of phlebotomy (>3 sessions/year) and increased risk of thrombosis in high-risk PV patients receiving HU [84]; however, this observation was not supported by a more powerful analysis of the aforementioned ECLAP and cytoreductive therapy in PV (CYTO-PV) database [83]. Two studies are noteworthy for their evaluation of thrombosis impact from *JAK2*V617F allele burden in PV [65, 82]; one included 320 patients [65] and found no association with thrombosis risk, although the authors reported an association with risk of fibrotic progression; the second study included 173 patients [82] and reported an association between *JAK2*V617F allele burden of >75% and cardiovascular events as well as increased need for cytoreductive therapy.

A recent systematic review and meta-analysis of articles involving over 30,000 patients suggested the role of leukocytosis in terms of arterial, but not venous, thrombosis in both PV and ET, although the association was stronger in the latter [80]. The methodology applied in this particular analysis was questioned by other investigators [85], who, in a subsequent report [64], did not find an association between persistent leukocytosis and thrombosis in PV; however, the latter analysis did not distinguish arterial from venous thrombosis because of low event rates [81]. The controversial contribution of leukocytosis, for thrombosis risk in PV, was also addressed in several other studies [78, 79, 86].

**Fig. 2:**
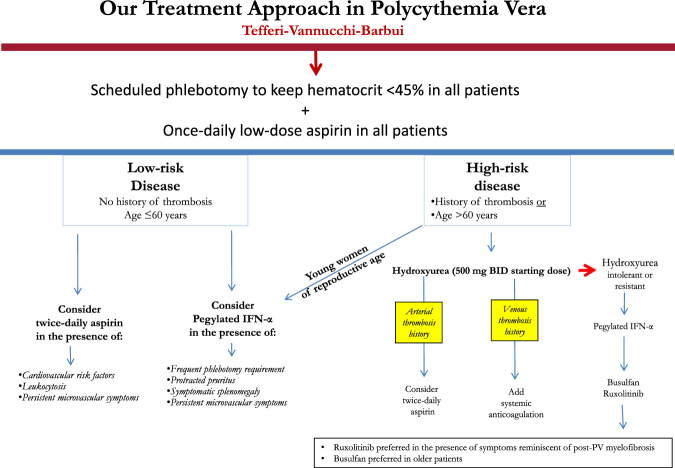
Current treatment approach in polycythemia vera. Our risk-adapted treatment algorithm in polycythemia vera (PV).

Based on the above outlined discussion, we consider history of arterial and venous thromboses in PV to be the most important risk factors for subsequent arterial or venous vascular events, respectively. We also endorse the inclusion of advanced age, variably defined as >60 or 65 years, as a major risk factor for both arterial and venous thrombosis. Cardiovascular risk factors signal the need for institution and dose optimization of aspirin therapy. At the present time, there is not adequate and reproducible evidence that allows formal inclusion of either leukocyte count or *JAK2*V617F allele burden, as independent risk factors for thrombosis in PV. As such, we do not use these variables to modify our overall treatment strategy (Fig. 2).

## How we treat

### Treatment backbone for all patients, regardless of risk category

The cornerstone of treatment in PV includes scheduled phlebotomy, with a Hct target of <45%, and daily low-dose aspirin therapy, in all patients, regardless of risk category [87]. The antithrombotic value of phlebotomy is supported by both controlled [39] and uncontrolled [33, 88] evidence and the case for phlebotomy in the treatment of PV was skillfully argued by William Dameshek in a 1968 commentary [15]. In a practically useful and elegant discussion, Barbui et al. considered the two most noteworthy controlled studies that are the basis for current recommendations regarding phlebotomy in PV [89, 90]; the PVSG-01 study [71] included 431 patients with PV who were randomized to receive phlebotomy alone or phlebotomy with P32 or chlorambucil; results of the study included respective median survivals of 13, 11, and 9 years; increased thrombosis risk in patients treated with phlebotomy alone, during the first 3 years; and increased rates of leukemic transformation and secondary cancers associated with chlorambucil or P32 treatment; the more recent CYTO-PV study included 365 patients with PV who were already receiving treatment with phlebotomy, HU, or both, prior to study entry, who were then randomly assigned to a target hematocrit goal of <45% or 45–50% [39]; after a median follow-up of 31 months, thrombotic events or deaths from cardiovascular causes were recorded in 5 of 182 patients in the <45% hematocrit group (2.7%) and 18 of 183 patients in the 45–50% hematocrit group (9.8%). These two studies provide the basis for current practice in terms of the need for phlebotomy and the desired Hct target in patients with PV. We are cognizant of limitations in both studies, which however do not undermine their overall value [89]. We are also receptive of situations where either the patient or their physician prefers a lower Hct target level (e.g., 42%) because of a variety of reasons including a lower baseline value for an individual patient (e.g., women vs men; in the setting of SVT or pregnancy) and the desire to achieve better symptom control or minimize excess residual risk of thrombosis despite standard therapy. In particular, a lower target Hct might be advisable during pregnancy since Hct levels are expected to be lower from the second trimester onwards

The therapeutic role of aspirin in the treatment of PV has long being suspected [71] and was initially faced with some concerns regarding bleeding complications [91], but its formal transition into routine clinical practice was facilitated by a controlled study from the ECLAP group [40]. The latter study enrolled 518 patients with PV in a double-blind randomized trial to low-dose aspirin (100 mg daily) or placebo [40]. Treatment with aspirin did not increase the incidence of major bleeding and instead reduced the risk of combined endpoints for “nonfatal myocardial infarction, nonfatal stroke, or death from cardiovascular causes” and “nonfatal myocardial infarction, nonfatal stroke, pulmonary embolism, major venous thrombosis, or death from cardiovascular causes” [92]. Aspirin therapy has also been reported, in a retrospective study, to be beneficial in *JAK2*V617F-mutated low-risk ET, in preventing venous thrombosis, and also in patients with cardiovascular risk factors, in preventing arterial thrombosis [93]. In addition to its value as an antithrombotic agent, low-dose aspirin therapy is also effective in alleviating microvascular symptoms in both PV and ET [94]. Recent studies have suggested greater antiplatelet effect from twice-daily, as opposed to once-daily dosing, not only in patients with MPN, but also in those with other diseases, where shortening of aspirin dosing interval is thought to overcome time-dependent renewal of the drug target [95]. In a recent meta-analysis of seven randomized clinical trials that included 379 participants [95], twice-daily vs once-daily aspirin dosing resulted in greater diminishing of serum thromboxane B2 (TxB2) levels, regardless of underlying disease phenotype. The particular phenomenon was specifically confirmed by several studies in the setting of MPN (mostly ET) [96–98], where increased platelet turnover is thought to compromise 24-h durability of aspirin inhibition of platelet cyclo-oxygenase-1; in one particularly noteworthy study, three aspirin dosing regimens (100 mg) were investigated and twice-daily/thrice-daily dosing was more effective, compared to once-daily dosing, in reducing platelet activation, measured by serum TxB2 level [97]. Although none of the aforementioned studies reported clinical outcome, their results were consistent in showing that multiple daily aspirin dosing was more effective than ASA 81 mg once-daily or 325 mg once-daily at suppressing serum TXB2, which is an in vivo marker of platelet activation. Regardless, controlled prospective studies are needed to confirm clinical relevance and safety of applying the specific treatment strategy in MPN as well as other diseases; in the meantime, we believe it is reasonable to consider twice-daily low-dose aspirin dosing in PV, in the absence of contraindications and presence of a higher risk for arterial thrombosis, including in patients with cardiovascular risk factors and leukocytosis. Aspirin therapy should be avoided in patients with bleeding symptoms associated with acquired von Willebrand syndrome; however, in the absence of bleeding history, we are comfortable in continuing with once-daily low-dose aspirin therapy provided ristocetin cofactor activity is above 20%, consistent with our current practice in ET [99].

### Indications and choice of cytoreductive drugs in low-risk PV

In general, cytoreductive therapy is not indicated for low-risk disease and its lack of additional value was indirectly surmised from a controlled study in ET [100]. However, aggressive phlebotomy in low-risk patients with PV might result in severe phlebotomy-induced side effects and might also not be adequate in controlling certain disease-associated symptoms such as severe pruritus [89, 101, 102]. As such, cytoreductive drugs might need to be considered, in addition to phlebotomy and aspirin, in such circumstances. In this regard, because guidance from high-quality controlled studies is currently lacking, one must rely on available experience from long-term prospective and retrospective studies and should always take patient preference into account, in deciding when to commence such treatment and which cytoreductive agent to choose [89, 101, 102]. Our first-line drugs of choice in the particular scenario include peg-IFN for younger patients and HU for older patients; our choices reflect consideration of the primary indication for treatment being control of symptoms or curbing the need for frequent phlebotomies, as opposed to prevention of thrombosis. Our proposed overall treatment strategy is supported by accumulating evidence of safety and treatment efficacy for peg-IFN, in the treatment of PV [103–108], its efficacy in alleviating intractable pruritus [109], and its selective but not consistent [110, 111] effect on the malignant clone [112–114]. A recent phase-2 randomized study of young patients (ages 18–60) with PV compared treatment with peg-IFN (ropeginterferon alfa-2b, 100 mg subcutaneous injection every 2 weeks) + phlebotomy + low-dose aspirin against phlebotomy + low-dose aspirin [115]; 127 patients were randomized to the two treatment arms and followed for a median of 12.1 months; the peg-IFN treatment arm provided superior hematocrit control (84% vs 60%) without significant difference between the two treatment arms in terms of grade 3 or higher adverse events. However, a numerically larger study with longer follow-up is needed, to clarify the role of cytoreductive therapy in low-risk PV; such a study will also address the growing debate on whether asymptomatic, low-risk PV patients should be treated with peg-IFN, outside of a clinical trial, on account of its selective anti-clonal activity and its anti-inflammatory properties.

### Management of high-risk disease

There is currently broad consensus regarding the need for cytoreductive therapy in high-risk patients with PV, in addition to phlebotomy and aspirin therapy [116]. There is also general agreement on which drugs should not be used in this regard (e.g., chlorambucil [71, 117], P32 [71], pipobroman [60, 118]) because of their previously well-demonstrated leukemogenic and/or carcinogenic potential. In contrast, a series of long-term prospective studies in PV [38, 41, 119–124] and randomized studies in ET [41, 125] have confirmed the favorable safety and efficacy record of HU therapy in high-risk disease. The pioneering study in this regard was a non-randomized PVSG trial that showed a lower incidence of early thrombosis in HU-treated patients, compared to a historical cohort treated with phlebotomy alone (6.6% vs 14% at 2 years) [38]. Similarly, the incidence of AML in the first 11 years of treatment was lower with HU, compared to historical controls treated with chlorambucil or P32 (5.9 % vs 10.6% vs 8.3%) [38]. Other studies have since confirmed the low incidence of AML in HU-treated PV (1–5.6%) [121, 126, 127].

In a recent reappraisal of 1042 PV patients from the ECLAP database, the authors reported an advantage for HU over phlebotomy alone, in terms of protection from fatal/nonfatal cardiovascular events and fibrotic transformation, whereas venous thrombosis rates were similar between the two treatment cohorts; leukemic transformation rate was very low (only three cases including two in the phlebotomy alone treatment group) after a median follow-up of approximately 3 years [124, 128]. A recent meta-analysis of 3236 HU-treated patients with PV [129] highlighted follow-up duration as an important variable in determining survival and fibrotic transformation and age in determining thrombosis rates (the latter were 1.9%, 3.6%, and 6.8% persons/year at median ages 60, 70, and 80 years, respectively). The particular study [129] estimated fibrotic transformation rates of 5% at 5 years and 34% at 10 years, while the respective mortality rates were approximately 13% and 56%; overall leukemic transformation incidence was lower at 0.4% persons/year and remained stable over time. In another ECLAP analysis of 1638 PV patients [130], which included 342 patients treated with phlebotomy alone and 700 treated in addition with HU, multivariable analysis did not disclose a significant difference in the risk of secondary malignancies other than AML, between the two treatment cohorts.

Favorable information regarding the therapeutic role of peg-IFN, in the setting of upfront therapy, is slowly accumulating and has now reached the point where it can be considered as an alternative option in young patients, although superiority or non-inferiority to HU is yet to be demonstrated in a controlled setting [42, 108, 131]. In a recent randomized study that compared pegylated INF (ropeginterferon alfa-2b; starting at 100 mcg subcutaneously every 2 weeks) with oral HU (starting at 500 mg/day), 257 PV patients with limited prior exposure to cytoreductive therapy were randomly assigned to one of the two treatment arms [42]; 217 patients completed the first part of the study and 171 patients were rolled over to the continuation part of the study. Complete hematological response with normal spleen size was achieved in 21% of patients receiving ropeginterferon vs 28% for HU, thus not meeting the criteria for non-inferiority. Similarly, hematologic response without meeting the spleen response criterion was similar between the two arms (43% vs 46%, respectively), at 12 months, as was the case with molecular response. In the extension part of the study, response rates, including molecular response, in patients receiving ropeginterferon gradually improved over time but with the difference not reaching statistical significance; however, at 3 years of treatment, significantly more patients receiving ropeginterferon maintained their hematological response. Treatment with ropeginterferon was associated with a spectrum of adverse events that required dose reductions in 40% of patients, dose interruption in 23%, and drug discontinuation because of drug-related toxicity in 8%. Common side effects of treatment with ropeginterferon included fatigue, liver function test abnormalities, thrombocytopenia, and leukopenia. Treatment-emergent serious adverse events were similar between the two treatment arms and follow-up was too short to evaluate differences in thrombosis or leukemic transformation rates. In a recent systematic review and meta-analysis of IFN (both peg and non-peg) treated patients with PV or ET [108], 44 studies including 1359 patients were analyzed; complete hematologic response rate in PV was reported at 49% with no difference between peg- and non-peg-IFN preparations; annualized rates of thrombotic complications and treatment discontinuation in patients with PV were estimated at 0.5% and 6.5%, respectively [108].

Taking the above-elaborated review and our own personal experiences into consideration, HU (starting dose 500 mg twice-daily) remains our current first-line cytoreductive drug of choice in older (age >60 years) patients with high-risk PV. We consider peg-IFN (starting dose 45 mcg weekly SC injection for pegasys or 100 mcg for every 2 weeks for ropeginterferon) as a reasonable alternative in younger patients and the preferred choice for young women of reproductive age and where treatment indication involves alleviation of symptoms (e.g., pruritus) or reducing the frequency of phlebotomies. Additional drug treatment choices (busulfan and ruxolitinib) are discussed below in the section of “treatment for HU refractory/intolerant PV.” Of note, neither prospective nor well-designed retrospective studies, in ET or PV, implicate HU as amplifying the intrinsic vulnerability of PV or ET patients for leukemic transformation [127, 132]. PV patients with venous thrombosis require systemic anticoagulation, in addition to cytoreductive drug therapy; we also consider adding low-dose aspirin in some instances in order to subvert the additional risk of arterial thrombosis, especially in the presence of *JAK2* mutation or cardiovascular risk factors, as well as lessen the risk of recurrence of venous thrombosis [77]. The therapeutic role of direct oral anticoagulants (DOACs) is currently being investigated and further elaborated below in the section of “management of splanchnic vein thrombosis.”

### Treatment of hydroxyurea refractory/intolerant PV

There are currently three drugs that are considered for use in patients who are intolerant or resistant to HU: pegylated IFN-α, ruxolitinib, and busulfan. Our first drug of choice in such an instance is peg-IFN. In a recent phase-2 clinical trial from the Myeloproliferative Disorders Research Consortium [133], 50 patients with PV and 65 with ET, who were refractory or intolerant to HU, received subcutaneous pegylated IFN-α (starting dose 45 mcg weekly and titrated to a maximum of 180 mcg) with 69% overall response rate, including 60% (22% complete response) in patients with PV. Statistically significant improvement in symptom burden was also noted but countered by pegylated IFN-α treatment-emergent adverse events, which were mostly tolerable; treatment discontinuation because of adverse events was relatively low at 14%. As expected, pegylated IFN-α therapy induced partial suppression of *JAK2*V617F in some patients [133]. Ruxolitinib (a JAK1/2 inhibitor) has also been shown to be effective in HU refractory/intolerant PV; in a phase-3 study (RESPONSE) comparing ruxolitinib (*n* = 110) with best available therapy (BAT; *n* = 112) [43], hematocrit/spleen control was achieved in 60%/40% of patients receiving ruxolitinib vs 20%/0.9% for BAT; 80-week follow-up [134] disclosed 83% of patients remaining on ruxolitinib therapy treatment, while 88% of the patients on BAT crossed over to ruxolitinib. Similar superiority in hematocrit control for ruxolitinib vs BAT (62% vs 19%) was shown in a subsequent randomized but not blinded study (RESPONSE-2) of PV patients without splenomegaly who need second-line therapy.

The efficacy of oral busulfan (dosed at 2–4 mg/day) in patients with advanced PV or ET refractory or intolerant to HU was assessed in two recent studies [135, 136]; in one study of 36 patients with treatment duration of 256 days, which included 15 patients with PV [135], complete hematological response was reported in 83% of the patients, which was sustained in the majority of the patients at 2 years. Busulfan was discontinued in 18 (67%) patients because of unmaintained remission, which is a unique feature of busulfan treatment response in patients with MPN; and 33% of informative cases demonstrated partial molecular response. The particular study listed 22% hematologic toxicity that was more likely to occur in patients receiving >14 mg/week [135]; with a follow-up of 117 person-years from initiation of treatment with busulfan, six patients had died, corresponding to a rate of 5.8 deaths × 100 person-years; causes of death were acute leukemia (*n* = 3), infection (*n* = 2), and unknown (*n* = 1). In addition, three cases of second neoplasms were reported, including cancers of the skin, prostate, and liver. The second study that included 51 informative patients with PV, a complete or partial hematologic response rate was reported in 75% of patients [136]; the study also reported a low (15%) rate of adverse drug effects and corresponding treatment dropouts (6%). Earlier studies of busulfan use in an upfront treatment setting, in both PV and ET, have also reported favorable efficacy and safety profile [137, 138]. On the other hand, the evidence for busulfan leukemogenicity in the context of treatment for PV or ET remains circumspective, and not validated in larger patient cohorts that accounted for other risk factors of leukemic transformation including older age, leukocytosis, and disease duration [60, 127, 139]; regardless, we are acutely aware and appreciative of opposing views on the subject matter and the possibility of increased risk for leukemic transformation, especially in patients receiving both busulfan and HU. One important confounding factor in the particular issue might involve busulfan dose and schedule; we recommend starting at the lower dose of 2 mg/day with close monitoring and consider periodic drug holidays, especially in the context of having achieved treatment objectives in controlling Hct and platelet count. Incidentally, drug-induced *JAK2*V617F allele burden reduction has also been demonstrated with busulfan use in PV [140].

Taking the above discussion into consideration, with emphasis on long-term track record of safety [104, 135, 141] and activity beyond symptom control (i.e., suppression of clonal myeloproliferation) [113, 114, 142], in patients who are either intolerant to or show suboptimal response to HU, we prefer the use of peg-IFN for patients younger than age 65 years and busulfan in the older age group, although there is no controlled evidence to support or refute such a strategy. Busulfan is started at 2–4 mg/day, withheld in the presence of platelets <200 × 10^9^/L or WBC < 3 × 10^9^/L, and the dose reduced to 2 mg/day when treatment is resumed after withholding. Ruxolitinib, on the other hand, is preferred in the presence of symptoms reminiscent of post-PV myelofibrosis and in patients suffering from drug-refractory pruritus or symptomatically enlarged spleen [43]. Whether or not ruxolitinib, peg-IFN, or busulfan provide protection from thrombosis, in addition to their salutary effect on hematocrit and other disease features, in resistant/intolerant PV, remains uncertain [143].

### Management during pregnancy

Reports of pregnancies in women with MPN are less common in PV than they are in ET, because, unlike the case with ET, PV has a male preponderance with only 15% of patients diagnosed before age 40 years [63]. Four relatively large studies reporting on pregnancies in PV included 8–48 patients with 5–121 pregnancies; live birth rates ranged from 61 to 88%, miscarriages from 13 to 29%, and maternal complications from 6 to 17% for thrombosis and 2 to 25% for bleeding [144–147]. Management included observation alone or treatment with aspirin, low molecular weight heparin (LMWH) or IFNα, alone or in combination; considering the retrospective nature of these studies, it is difficult to discern the specific circumstances or treatments that might have influenced outcome. Regardless, we highly recommend preconceptual counseling regarding risk of fetal loss and other complications, especially in patients with prior pregnancy loss or history of thrombosis. Along with strict control of Hct < 45% (preferably below 42%), treatment with low-dose aspirin is recommended in all PV patients planning to be pregnant, based on favorable observations extrapolated from the experience in ET, regarding protection from first trimester fetal losses [148]. We recommend cytoreductive therapy with peg-IFN in patients with prior vascular events and consider adding LMWH, in case of venous thrombosis history; we do not advise the use of HU or warfarin because of their teratogenic potential. The value of LMWH during pregnancy or post-partum, in the absence of venous thrombosis history, is uncertain.

### Management of splanchnic vein thrombosis

Although it is well known that SVT frequents patients with MPN, including MPN-U, its optimal management remains obscure. As a background on SVT in general, in one population-based study of 1915 patients [149], the affected veins were portal in 78%, hepatic in 11%, and mesenteric in 11%; risk was similar between the two sexes and the respective incidence rates were 21, 3, and 3/100,000 persons per year. In the study [149], comorbidities included recent surgery (40%), liver cirrhosis (11%), pancreatitis (11%), gastrointestinal cancer (9%), extraintestinal cancer (10%), and MPN (1.2%). The incidence of MPN as a comorbid condition was higher in another study (8%) [150]. In a recent retrospective study, 518 patients with MPN-SVT were compared to 1628 otherwise unselected MPN cases [151]; the former were more likely to be younger, females, and *JAK2*V617F mutated (90%). The study included 192 (37%) patients with PV (median age 45 years; 53% females) and 178 (34%) with ET (median age 39 years; 71% females; 85% *JAK2* mutated) and affected veins included portal (67%), hepatic (25%), splenic (29%), and mesenteric (24%) [151]. A concomitant hypercoagulable disorder was documented in 39% of the cases. SVT recurrence rate was 1.6 per 100 patient-years and significantly improved by treatment with vitamin K-antagonists (VKA) but not cytoreductive therapy. Bleeding complications did not appear to be influenced by VKA therapy but were more likely to occur in patients with esophageal varices. Overall survival of PV patients in the study [151] was not affected by SVT; furthermore, there was little evidence of disease progression in patients with MPN-U with SVT (*n* = 55). Other studies have confirmed lower *JAK2*V617F allele burden, lower blood counts higher likelihood of concomitant hypercoagulable state, higher risk of venous thrombosis, and bleeding, in MPN-SVT, compared to their MPN counterparts without SVT [152, 153]. A more recent, similarly retrospective report looked into risk factors for adverse outcome in 80 patients with MPN-SVT (mostly PV) [154]; at a median follow-up of 11 years, 13% of the patients experienced an adverse outcome and were enriched for cases with ≥50% *JAK2*V617F allele burden, and additional mutations (spliceosome or *TP53*); MPN-SVT patients with at least one of the latter two risk factors displayed inferior event-free (81% vs 100%) and overall (89% vs 100%) survival at 10 years.

Taking the above observations and those of other studies into account [153, 155], it is reasonable to state that survival in MPN-SVT is primarily influenced by that of the underlying MPN, rather than the SVT event itself, which would be consistent with the observation regarding shortened survival in patients with abnormal karyotype [155] or certain high-risk mutations [154]. Currently, there are not reliable predictors of first-event or recurrent SVT in MPN, including PV. The therapeutic value of systemic anticoagulation (and the choice between VKA and DOAC) [151] or cytoreduction (and the choice between HU and IFN) [156, 157] requires further validation, in a controlled setting. In a small study of ruxolitinib, therapy in patients with MPN-SVT did not indicate salutary effect on esophageal varices or mesenteric circulation [158], which is consistent with lack of evidence for its value in reducing thrombosis risk [143]. In general, in patients with PV/MPN-associated SVT, we recommend an aggressive treatment approach that includes, at a minimum, systemic anticoagulation [156, 157]; in the latter regard, we consider patient preference and convenience regarding the choice between VKA and DOACs, since we are not yet convinced that one is better than the other; regardless, we prefer LMWH therapy in the acute setting followed by VKA therapy, especially if there is concern regarding intestinal edema or variceal bleeding associated with portal hypertension; we believe DOAC therapy is a reasonable alternative, otherwise.

### Perioperative management

It is important to consider the possibility of increased risk of thrombosis or hemorrhage in PV patients undergoing surgery, in lieu of their underlying *JAK2*-mutated MPN as well as the generally expected post-surgical risk of thrombosis and bleeding. There are currently limited data for guidance regarding optimal pre- and perioperative management of patients with PV or ET. In a 1963 report by Wasserman and Gilbert [159], 62 major surgical operations in patients with PV were analyzed and revealed fatal and nonfatal complication rates of 83% vs 21%, in hematologically uncontrolled vs controlled disease, respectively. More recent studies have suggested more favorable outcome. In one such study [160], 255 patients with PV or ET were analyzed for a total of 311 surgical interventions, including 25 emergency procedures. Antithrombotic prophylaxis included subcutaneous heparin in 54% and antiplatelet therapy in 15% of the patients. In addition, 74% of patients were on cytoreductive therapy before surgery. Three-month post-operative course was uneventful in more than 80% of the cases, whereas arterial or venous events were documented in 12 patients, each, with the former being more frequent in ET and the latter in PV; major bleeding complications occurred in 23 cases and deaths in 5; platelet count and hematocrit level at time of surgery were not predictive of vascular events and the value of pre-procedure prophylactic therapy was not apparent. Regardless, our current practice is based more on intuition rather than evidence and includes keeping hematocrit level below 45% and platelet count below 450 × 10^9^/L, before and after surgery; platelet count control in low-risk patients might require a short course of treatment with HU; in addition to cytoreductive therapy, careful use of LMWH is advised in high-risk patients.

### Management of pruritus

Pruritus is a particularly vexing symptom associated with PV and is often exacerbated by contact with water [161–163]. In a large cohort of 441 German patients with PV [163], patient-directed questionnaire revealed that 68% of the patients were affected by aquagenic pruritus, in the majority occurring before formal diagnosis of PV; pruritus manifested in different forms including itching, tickling, stinging, or burning sensations and its severity was labeled unbearable in 15% of the cases. In a review of the literature spanning the period 1965–2009 [162], application of a variety of treatment modalities, including antihistamines, antidepressants, IFN-α, phlebotomy, phototherapy, iron supplements, and myelosuppressive medications, was documented with mixed efficacy results. In the low-risk disease setting, we first consider non-drug measures, such as avoidance of precipitating conditions, dry skin, and temperature control of one’s environment and water used for bathing. In general, treatment responses to antihistamines have been both unpredictable and variable [161]. More favorable responses have been reported with use of selective serotonin reuptake inhibitors [164] and narrow-band ultraviolet B phototherapy [165]. In high-risk disease setting, both *JAK2* inhibitors [166, 167] and IFN-a [168] have shown therapeutic activity, which is not shared by HU.

### Management of post-PV myelofibrosis

In the absence of genetic information other than *JAK2*, it is current practice to stratify patients with post-PV MF in a similar fashion to those with PMF, based on a previously published demonstration of similar applicability for risk models used for the latter, including IPSS, DIPSS, and DIPSS-plus [169]. The suboptimal performance of these clinical models has been addressed by more contemporary mutation-enhanced prognostic models [170, 171]. For example, MYSEC-PM (Myelofibrosis Secondary to PV and ET-Prognostic Model) [171] considers constitutional symptoms, anemia, circulating blasts, thrombocytopenia, advanced age, circulating blasts, and absence of *CALR* mutations as risk variables. One can also consider other mutation-enhanced models derived in the setting of PMF, including MIPSS70 [172] and MIPSSv2 [173, 174], which in addition consider karyotype and high-risk mutations, including *ASXL1*, *SRSF2*, *EZH2*, *IDH1*/*IDH2*, and *U2AF1*Q157 [171, 173–176]. From a practical standpoint, we believe that both MIPSS/MIPSSv2 and MYSEC adequately serve their main purpose in identifying high-risk patients with post-PV MF who should be referred for allogeneic hematopoietic stem cell transplant sooner rather than later. Non-transplant therapies for post-PV MF are like those for PMF and mostly palliative [177].

## New drugs in the horizon

### PTG-300 (hepcidin mimetic)

PTG-300 (Rusfertide) is a hepcidin mimetic whose mechanism of action includes restriction of iron availability (i.e., negative iron regulation) for red blood cell production. In other words, it recapitulates iron deficiency at the cellular level, without depleting iron stores. The drug is administered by weekly subcutaneous injection at escalating doses of 10, 20, 40, 60, and 80 mg, adjusted to maintain hematocrit <45%. The most recent (2020) EHA presentation abstract included 35 patients including 16 with low-risk disease [178]. PTG-300 therapy resulted in significant reduction of phlebotomy need; among 13 patients treated for at least 28 weeks, 10 remained phlebotomy free and concomitant iron deficiency was reversed in most instances, associated with improvement in symptoms. Reported side effects of PTG-300 included transient low-grade injection site reactions. Of note, PTG-300 did not appear to affect leukocyte or platelet count [178]. We are not certain about the prospect of PTG-300 within the therapeutic program for PV for a number of reasons; first, high-risk patients require broader myelosuppression to prevent thrombotic complications and such treatment is often more than adequate to control the hematocrit as well; second, in low-risk patients, hematocrit control is easily achieved with drug-free phlebotomy and if an alternative to phlebotomy is needed, for one reason or another, peg-IFN offers a more attractive option since it also controls thrombocytosis, leukocytosis, splenomegaly, and certain symptoms such as aquagenic pruritus. Also, peg-IFN has a much longer track record of safety.

### Idasanutlin (MDM2 antagonist)

Idasanutlin is an orally administered (150 mg once-daily × 5 repeated every month) Mouse Double Minute 2 (MDM2) antagonist whose mechanism of action includes stabilization of TP53 activity by blocking its binding to MDM2. In a phase-2 trial of 27 phlebotomy-dependent patients with PV who were resistant or intolerant to HU therapy, patients were treated for a median of 257 days; hematocrit control was achieved in 9 (56%) patients, complete hematologic response in 8 (50%), spleen volume response in 7 (33%), and symptoms response in 6 (43%) [179]. In addition, 76% of patients who were evaluable at week 32 of treatment experienced reduction in *JAK2* mutant allele burden. Unfortunately, most patients experienced significant gastrointestinal toxicity including nausea, vomiting, and diarrhea, which contributed to the need for dose modifications, documented in 63% of patients. These concerns are further compounded by the possibility of treatment-emergent expansion of mutant *TP53* clones [180], making it unlikely for the drug to garner continued interest in the treatment of PV.

### Givinostat (HDAC inhibitor)

Givinostat is a histone-deacetylase (HDAC) inhibitor that selectively targets *JAK2*-mutated clones. In a series of early phase studies, givinostat was administered orally (50–100 mg BID) in 50 patients with PV, either alone or in combination with HU (*n* = 15) [181]. At the time of the most recent analysis on long-term outcome, median drug exposure was 2.8 years and 62% of the patients remained on active therapy. Treatment-emergent adverse effects (26% considered serious) of givinostat included QTc prolongation, thrombocytopenia, diarrhea, dysgeusia, and headache. Givinostat-treated PV patients benefitted the most in terms of alleviation of pruritus and control of blood counts, including hematocrit, while the drug had limited activity in reducing spleen size; in some instances, clinical response was accompanied by reduction in *JAK2* mutant allele burden [181]. Results from a planned phase-3 trial are awaited, in order to position givinostat in either upfront or second-line therapy in PV.

## Concluding remarks

Although we are comforted with the relatively indolent clinical course of patients with PV [63, 70], we are acutely aware of outstanding issues including residual risk of thrombosis despite “optimal” current therapy, impaired quality of life from frequent phlebotomy needs or non-thrombotic symptoms in some patients, and the apparently inevitable risk of premature death and/or disease transformation into AML or post-PV MF. Regarding the latter, currently available therapy has not been shown to modify the natural history of the disease and clinical trials addressing the issue are challenged by the need for a controlled setting and long-term follow-up. We acknowledge the possibility of drug-induced suppression of *JAK2*V617F allele burden, seen in some patients treated with peg-IFN [103] or busulfan [140], but question its translation into longer survival or decreased risk of disease transformation; furthermore, there appears to be limited correlation between molecular and hematologic response [142]. On the other hand, we believe that there is feasibility for productive clinical trials directed at maximizing thrombosis protection and improving quality of life, as long as such studies incorporate careful monitoring for long-term assessment of untoward drug effects. In this regard, the currently most attractive candidate drug is peg-IFN, whose therapeutic value and safety profile has been studied in a controlled setting, in both low [115] and high [42] risk PV, although follow-up time was too short to allow making definitive conclusions. We believe that larger scale studies involving peg-IFN are warranted to clarify value in low-risk patients with intent to curb phlebotomy requirements and alleviate non-thrombotic symptoms [115]. Finally, the possibility of maximizing thrombosis protection with twice- vs once-daily aspirin therapy or use of DOACs should be pursued in future clinical trials; the added value of the former has been suggested by its greater antiplatelet effect [95–97] and the latter in recent descriptive studies [182–185]. Of note, the concomitant use of aspirin and DOAC was recently associated with excessive bleeding without additional value in thrombosis prevention, in patients with atrial fibrillation or venous thromboembolic disease [186]; whether or not this holds true in the context of PV remains to be studied.
